# Checking Wilderness Through Conservation Genetics: The Case of the European Wildcat in the Central Apennines, Italy

**DOI:** 10.1002/ece3.74418

**Published:** 2026-09-24

**Authors:** Melissa Maini, Chiara Polce, Francesca Bernini, Maria Longeri, Raffaella Milanesi, Romolo Caniglia, Federica Mattucci, Edoardo Velli, Roberto Cazzolla Gatti

**Affiliations:** ^1^ Department of Biological, Geological and Environmental Sciences Alma Mater Studiorum University of Bologna Bologna Italy; ^2^ Independent Consultant Varese Italy; ^3^ Department of Veterinary Medicine and Animal Sciences University of Milan Lodi Italy; ^4^ Unit for Conservation Genetics (BIO‐CGE) Italian Institute for Environmental Protection and Research (ISPRA) Ozzano dell'Emilia Italy

**Keywords:** conservation strategies, environmental predictors, European wildcat, *Felis silvestris silvestris*, genetic diversity, species distribution model

## Abstract

The European wildcat (*Felis silvestris silvestris*, EWC) is threatened by habitat fragmentation, hybridisation with domestic cats, and climate change, yet its distribution and genetic status in Italy remain poorly resolved. Here, we developed a species distribution model (SDM) based on 69 occurrence records compiled from published sources to identify suitable habitats across the Italian peninsula and characterised 23 newly collected occurrences from the Central–Southern Apennines, which included associated biological samples. The model identified forest and grassland cover and elevation as the main predictors of occurrence, with high suitability concentrated in the Apennine and Alpine regions. Most newly collected occurrences fell within areas of high predicted suitability. Given the potential phenotypic overlap among wildcats, domestic cats, and admixed individuals, we complemented the ecological assessment with mitochondrial DNA (MT‐ND5) analysis to characterise the maternal lineages of phenotypically identified wildcats and assess their correspondence with previously described wildcat and domestic cat haplotypes. Mitochondrial analysis (MT‐ND5) revealed that 85% of individuals carried haplotypes shared between wildcats and domestic cats, whereas only 10% carried haplotypes previously reported exclusively in wildcats. These patterns indicate a predominance of shared maternal lineages, but given the limitations of mtDNA, they cannot alone be interpreted as evidence of contemporary hybridisation. Our results provide a first country‐wide assessment of habitat suitability for the EWC in Italy and highlight the value of integrating ecological modelling with empirical occurrence and genetic data to support conservation planning.

## Graphical abstract

We developed a species distribution model to assess habitat suitability for the European wildcat across Italy, integrating newly collected occurrence records and mitochondrial DNA data from the Central–Southern Apennines. Forest and grassland cover and elevation emerged as the main predictors of occurrence, with the highest suitability concentrated in the Apennine and Alpine regions. Mitochondrial analyses revealed a predominance of shared maternal lineages, highlighting the value of integrating ecological modelling, occurrence data and genetic information to support wildcat monitoring and conservation planning.

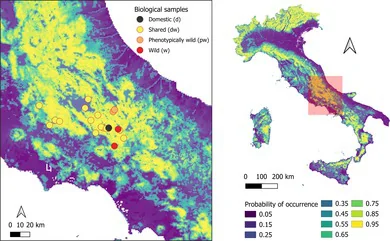

## 1 Introduction

Knowledge of the distribution of an endangered wildlife species is crucial for implementing effective conservation measures (Lancia et al. 1994; Gil‐Sánchez et al. 2020). However, such information is often lacking or incomplete. A representative example is the European wildcat ( *Felis silvestris silvestris* , Schreber 1777, hereafter EWC), a medium‐sized felid distributed heterogeneously throughout Europe, from the Iberian Peninsula to the Caucasus and as far north as Scotland. Land‐use changes caused by anthropogenic activities threaten this species through habitat fragmentation and disturbances (e.g., tourist presence in previously inaccessible areas), roadkill mortality, and potential hybridisation with domestic cats (Lozano and Malo 2012). Although globally classified as ‘Least Concern (LC)’ by the IUCN (Gerngross et al. 2022), the EWC is a species of conservation concern in Europe. It is listed in Annex IV of the European Habitats Directive (92/43/EEC) as a ‘strictly protected’ species and included in Appendix II of both CITES and the Bern Convention (Lozano and Malo 2012). Furthermore, in Italy, the EWC is strictly protected under Hunting Law n. 157/92 and Law n. 357/97.

In Italy, knowledge of EWC occurrence has progressively increased through local and regional ecological studies and monitoring initiatives (Anile et al. 2017, 2019, 2021; Lazzeri et al. 2022; Ferretti et al. 2022; Viviani et al. 2024). Available records indicate a discontinuous distribution encompassing the Alpine region, the Apennine chain, and Sicily. Previous studies have shown that EWC occurrence is associated with structurally complex natural habitats, particularly shrublands and mixed or forested habitats (Lozano 2010; Anile et al. 2019; Lazzeri et al. 2022). Shrub‐dominated habitats may provide favourable conditions for shelter, protection, and prey availability, whereas forest cover and landscape structure are important components of wildcat habitat use (Lozano 2010; Ruiz‐Villar et al. 2023). Conversely, habitat fragmentation and increasing anthropogenic pressure can negatively affect wildcat occurrence and space use (Anile et al. 2019, 2021; Ruiz‐Villar et al. 2023). The species is predominantly nocturnal and elusive, with activity patterns that may vary in relation to environmental conditions and human disturbance (Lazzeri et al. 2022; Anile et al. 2021). These ecological and behavioural characteristics can hamper detection and contribute to incomplete knowledge of the distribution of this species.

Despite these advances in knowledge of EWC occurrence, existing studies in Italy have largely focused on specific geographical areas or particular aspects of wildcat ecology, and a spatially explicit, country‐wide assessment of habitat suitability remains lacking. This gap is particularly relevant in the Central–Southern Apennines, where occurrence information remains incomplete despite the presence of potentially suitable habitats.

With the support of Rewilding Apennines (RA) as part of the LIFE ESC360 project (LIFE17 ESC/IT/000001), we aimed to address this knowledge gap by predicting suitable areas for the EWC in Italy and increasing the number of occurrence records to improve the description of its distribution in the Central‐Southern Apennines.

To identify areas potentially suitable for the EWC, we developed a species distribution model (SDM) using all available wildcat occurrence records in Italy at the time of the study, the results of which were also used to characterise the environment of newly collected occurrences from the Central–Southern Apennines.

These new occurrences were associated with biological samples from individuals phenotypically identified as putative EWCs through camera‐trap footage and morphological characteristics, including pelage traits consistent with those described as diagnostic for wildcat identification (Ragni and Possenti 1996; Kitchener et al. 2005). However, given the potential phenotypic overlap among wildcats, domestic cats, and admixed individuals, these assignments were considered putative rather than definitive taxonomic identifications. To complement the ecological assessment of the new occurrences, mitochondrial DNA variability was analysed to characterise maternal lineages and compare the detected haplotypes with those previously described in wildcats and domestic cats from Europe and Italy (Velli et al. 2023).

Overall, this integrative approach aimed to provide a spatially explicit assessment of EWC distribution and habitat suitability in Italy, with particular attention to the poorly investigated Central–Southern Apennines, together with preliminary information on the maternal genetic composition of sampled individuals.

## 2 Materials and Methods

### 2.1 Species Distribution Model

#### 2.1.1 Area and Occurrence Data

Italy encompasses a wide range of ecosystems, spanning from Alpine environments in the north to Mediterranean landscapes in the south. Its physiographic heterogeneity—which includes mountains, forests, wetlands, and coastal systems—supports a high level of biodiversity and numerous endemic taxa, making Italy one of the most species‐rich countries in Europe (Labra 2019; Garofalo and Ingrassia 2024).

**FIGURE 1:**
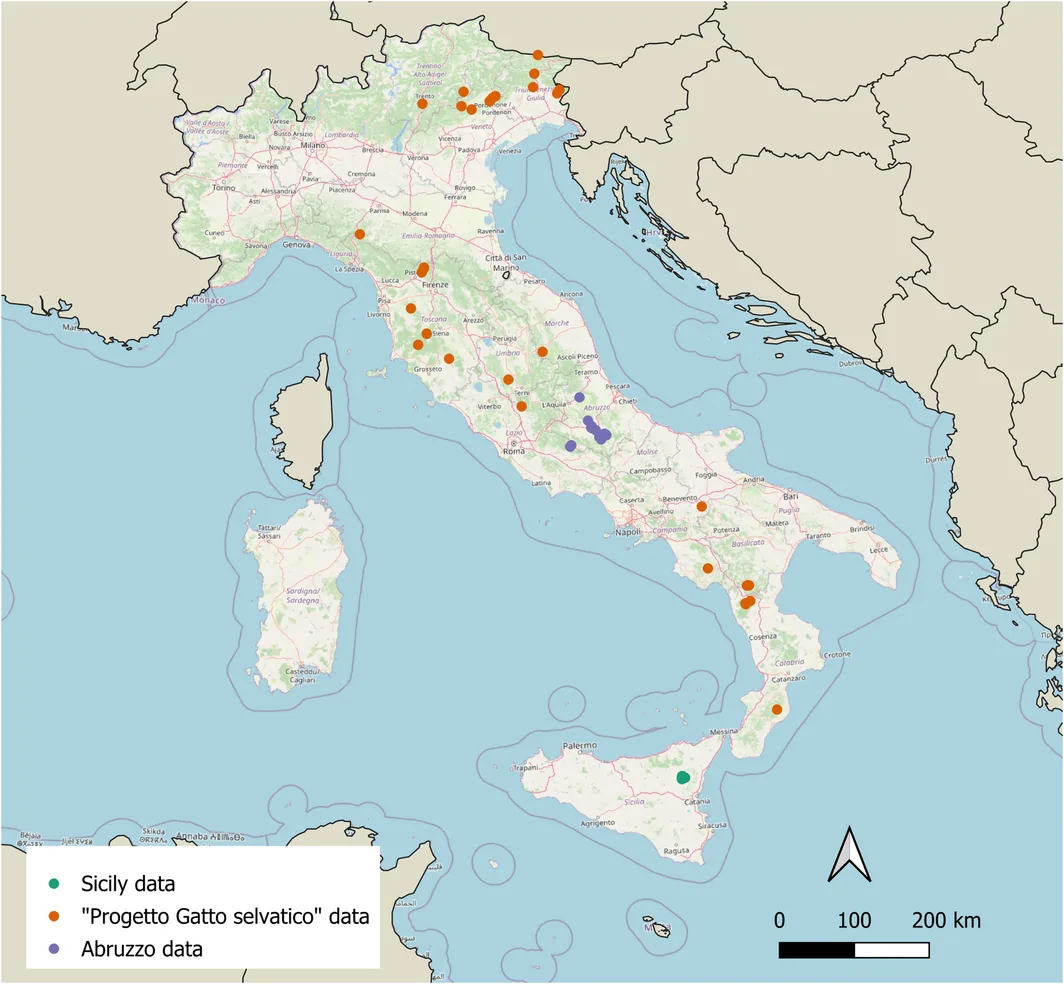
The 69 spatially explicit European wildcat records, with dot colours indicating the source: Progetto Gatto Selvatico (orange), Abruzzo, RA (purple), and Sicily, Anile et al. 2012 (green). Basemap: OpenStreetMap contributors, available under the Open Database Licence. Obtained through the QuickMapServices plugin in QGIS.

We compiled 69 spatially explicit occurrence records of *Felis silvestris silvestris* from three independent datasets to develop our distribution model. The 69 occurrence records covered the period 2009–2023. Their temporal distribution was concentrated around the reference year of the land‐cover data, with 54 records (78.3%) collected between 2013 and 2023, i. e., within ±5 years of the CORINE Land Cover 2018 reference year. Occurrence points were collected from northeastern Friuli to southern Sicily, covering a mosaic of natural habitats inside and outside protected areas and national parks. Occurrence points were obtained from the following sources and locations (Figure 1; Table S1):

- *n* = 35 records from Friuli‐Venezia Giulia (UD), Trentino (TN), Veneto (BL), Emilia‐Romagna (PR), Tuscany (FI, PO, SI, GR), Marche (MC), Lazio (RI), Campania (AV, SA), Basilicata (PZ), and Calabria (CS, RC). These records, derived from camera‐trapping surveys and carcass recoveries, were provided by the ‘Progetto Gatto Selvatico’ and made available through the Natural History Museum of Maremma (https://www.museonaturalemaremma.it/gatto‐selvatico‐italia/). The project was developed to consolidate national EWC data in collaboration with ISPRA and the Italian Ministry for the Environment and Energy Security.
- *n* = 22 records from Abruzzo (AQ), obtained through camera‐trapping activities conducted by RA as part of the LIFE ESC360 project (LIFE17 ESC/IT/000001). These data are released and used here for the first time.
- *n* = 12 records from Sicily (CT, Etna Regional Park), collected through camera‐trapping surveys reported in Anile et al. (2012).

To maximise data reliability, the 35 records obtained from Progetto Gatto Selvatico were classified according to the species‐specific SCALP scheme, originally developed for large‐carnivore monitoring (Molinari‐Jobin et al. 2012) and subsequently adapted to the European wildcat (Gerngross et al. 2023). Under this species‐specific classification, C1 records are supported by reliable molecular‐genetic evidence, whereas C2 records are based on verifiable and confirmed morphological or phenotypic evidence, including photographs or videos showing diagnostic wildcat characteristics. The classification of the 35 records from Progetto Gatto Selvatico resulted in 11 C1 and 24 C2 records. The 11 C1 occurrences were supported by both morphological and genetic evidence, including individuals recorded via camera traps and confirmed by either samples or road‐killed carcasses. The 24 C2 occurrences were associated with unequivocal camera‐trap photographs of phenotypically wild‐type individuals.

All 69 occurrence records were georeferenced using their geographic coordinates, which were used as point‐presence data for model calibration and mapped using QGIS 3.28.14 (Figure 1).

#### 2.1.2 Environmental Predictors

We selected two categories of environmental predictors to model EWC distribution across Italy:

1. Land Use/Land Cover (LULC). LULC classes from CORINE Land Cover 2018 (Version 2020_20u1; 100 m resolution) were extracted at hierarchical Level 3 and aggregated to eight classes (continuous and discontinuous urban fabric, road and rail networks, pastures and agriculture, fruit trees and olive groves, agroforestry, forest and grassland, scrubland and sparsely vegetated land, water courses and water bodies) to reflect cover types relevant to EWCs. Each LULC class was converted to percent cover within 1‐km grid cells, thus resulting in eight continuous variables (Table 1).
2. Topography. Average elevation per 1‐km grid cell derived from the SRTM Digital Elevation Model (90 m resolution; NASA 2000).

**TABLE 1:** Description of the land use/land cover (LULC) predictors used in the analysis, with correspondence to the original CORINE Land Cover classes.

LULC predictor	LULC code	CORINE LC Level 3 class	CORINE LC Level 3 code	CORINE LC grid code
Continuous and discontinuous urban fabric	L1_UF	Continuous urban fabric	111	1
		Discontinuous urban fabric	112	2
		Industrial or commercial units	121	3
		Construction sites	133	9
		Green urban areas	141	10
Road and rail networks	L2_RR	Road and rail networks and associated land	122	4
Pastures and agriculture	L3_PAG	Non‐irrigated arable land	211	12
		Permanently irrigated land	212	13
		Rice fields	213	14
		Vineyards	221	15
		Pastures	231	18
		Annual crops associated with permanent crops	241	19
		Complex cultivation patterns	242	20
		Land principally occupied by agriculture, with significant areas of natural vegetation	243	21
Fruit trees and olive groves	L4_FTO	Fruit trees and berry plantations	222	16
		Olive groves	223	17
Agro‐forestry	L5_AGF	Agro‐forestry areas	244	22
Forest and grassland	L6_F	Broad‐leaved forest	311	23
		Coniferous forest	312	24
		Mixed forest	313	25
		Natural grasslands	321	26
		Sclerophyllous vegetation	323	28
Scrubland and sparsely vegetated land	L7_SSV	Moors and heathland	322	27
		Transitional woodland‐shrub	324	29
		Sparsely vegetated areas	333	32
Water courses and water bodies	L8_WCB	Intertidal flats	423	39
		Water courses	511	40
		Water bodies	512	41
		Estuaries	522	43

Regarding temporal correspondence, CORINE Land Cover 2018 falls within the temporal range of the occurrence dataset and was used as a representative land‐cover reference for most occurrence records. Elevation derived from the SRTM Digital Elevation Model was considered effectively invariant over the temporal scale examined.

All spatial analyses were conducted in QGIS 3.28.14 (2022), using the European Lambert Azimuthal Equal‐Area (EPSG:3035) projection.

The choice of predictors was confirmed after testing for collinearity (Spearman's correlation > |0.7| was adopted as a cut‐off point to exclude variables). Given the relatively small number of occurrence records, we deliberately retained a parsimonious set of predictors to reduce model complexity and limit the risk of overfitting, while prioritising overarching land‐cover and topographic variables consistently identified as ecologically relevant to wildcat distribution in previous studies.

#### 2.1.3 Model Settings and Evaluation (MaxEnt)

Species distribution modelling was performed using MaxEnt 3.4.3 (Phillips and Schapire 2004), suitable for presence‐only data. Given the relatively small, presence‐only dataset and the spatially heterogeneous sampling effort, we adopted a single, explicitly tuned modelling framework (MaxEnt) to retain model parsimony while allowing sampling bias and model complexity to be directly controlled. To test and eventually account for uneven sampling effort across the study area (Phillips et al. 2009), we created a two‐dimensional kernel density estimate of occurrence localities using the kde2d() function from the MASS package in R (Venables and Ripley 2002). The function generates a continuous surface representing relative sampling effort, which is used to weight background points (the pseudo‐absences) so that the selection mimics the same sampling biases as the presence data (Phillips et al. 2009; Inman et al. 2021). This bias file was used to generate 10,000 background points (pseudo‐absences). Model tuning was conducted with ENMevaluate() in the R package ENMeval 2.0.4 (Muscarella et al. 2014). The function fits models across a grid of regularisation multipliers (rm) and feature classes (fc), which control model complexity and response shapes. Occurrences were partitioned using 10 random k‐folds (partitions = ‘randomkfold’) and candidate models were ranked by the Akaike Information Criterion. The optimal model (ΔAIC = 0) included hinge features and a regularisation multiplier (rm) of 3; these settings were used for the final Maxent model. We chose ‘cloglog’ for the output format, which approximates the probability of occurrence (Phillips et al. 2017).

Model performance was evaluated using the Area Under the Curve (AUC), acknowledging its limitations for presence‐only models (Austin 2007; Termansen et al. 2006; Wiley et al. 2003). To assess whether the observed AUC exceeded values expected under a null model, the empirical AUC (AUC_SDM_) was compared with a distribution of null model AUCs (AUC_NM_). This distribution was obtained from 100 iterations generated using ENMnulls (Raes and Ter Steege 2007), using the same spatial partitions (Bohl et al. 2019; Kass et al. 2020).

### 2.2 New Occurrences With Biological Samples

#### 2.2.1 Area and Occurrence Data

A total of 23 new EWC occurrences from the Central–Southern Apennines were included in this study (Table 2). None of these 23 occurrences was included among the 69 occurrence records used for SDM calibration. Sixteen tissue samples (muscle or skin) were obtained from carcasses collected in the Abruzzo, Lazio and Molise National Park (*n* = 11) and the Regional Natural Park of the Simbruini Mountains (*n* = 5). These carcasses, mostly resulting from roadkill mortality and, in a few cases, accidental hunting bycatch, were recovered by park rangers and submitted to the Istituto Zooprofilattico dell'Abruzzo e Molise for necropsy and tissue sampling. Seven additional occurrences were obtained through non‐invasive monitoring activities combining camera traps and hair traps, carried out by Rewilding Apennines (RA) within the LIFE ESC360 citizen‐science programme, in collaboration with the Carabinieri of Castel di Sangro (AQ). Five of these occurrences were recorded in the Monte Genzana–Alto Gizio Regional Nature Reserve and two in the Sirente–Velino Regional Natural Park and adjacent areas, with hair samples collected from all seven individuals. For one of the two individuals recorded in the Sirente–Velino area (RA3), a muscle sample was also available and analysed.

**TABLE 2:** Summary of the new occurrences with biological samples collected in the Central‐Southern Apennines.

Sample name	Municipality	Protected areas and parks	Longitude	Latitude	Date	Sample type
SMP1	Vallepietra (FR)	Regional Natural Park of the Simbruini Mountains	13,206902	41,87538	19/02/2009	Muscle
SMP2	Piglio (FR)	Bordering Regional Natural Park of the Simbruini Mountains	13,135313	41,83033	21/12/2016	Muscle
SMP3	Arsoli (RM)	Regional Natural Park of the Simbruini Mountains	13,016859	42,01671	29/12/2019	Muscle
SMP4	Trevi nel Lazio (FR)	Regional Natural Park of the Simbruini Mountains	13,26058	41,86264	11/03/2022	Muscle
ALMP1	Pizzone (IS)	Abruzzo, Lazio and Molise National Park	14,03794	41,67827	11/06/2014	Muscle
ALMP2	Barrea (AQ)	Abruzzo, Lazio and Molise National Park	13,992848	41,75576	22/10/2014	Muscle
ALMP3	Pescasseroli (AQ)	Abruzzo, Lazio and Molise National Park	13,794222	41,78295	11/02/2015	Muscle
ALMP4	Pescasseroli (AQ)	Abruzzo, Lazio and Molise National Park	13,768442	41,79352	26/01/2016	Skin
ALMP5	Gioia Vecchio, Gioia dei Marsi (AQ)	Abruzzo, Lazio and Molise National Park	13,733669	41,90374	26/01/2017	Muscle
ALMP6	Villavallelonga (AQ)	Abruzzo, Lazio and Molise National Park	13,646017	41,85850	16/01/2018	Muscle
ALMP7	Opi (AQ)	Abruzzo, Lazio and Molise National Park	13,855759	41,77552	04/01/2018	Muscle
ALMP8	Campoli Appennino (FR)	Abruzzo, Lazio and Molise National Park	13,696050	41,74026	01/12/2018	Muscle
ALMP9	Barrea (AQ)	Abruzzo, Lazio and Molise National Park	13,974908	41,76412	30/01/2019	Muscle
ALMP10	Opi (AQ)	Abruzzo, Lazio and Molise National Park	13,829385	41,77558	14/12/2021	Muscle
ALMP11	Opi (AQ)	Abruzzo, Lazio and Molise National Park	13,859802	41,78035	16/12/2021	Muscle
ALMP12	San Biagio Saracinisco (FR)	Abruzzo, Lazio and Molise National Park	13,919414	41,61077	06/04/2022	Muscle
RA1	Pettorano sul Gizio (AQ)	Monte Genzana – Alto Gizio Regional Nature Reserve	13,942786	41,94632	19/04/2021	Hair
RA2	Pettorano sul Gizio (AQ)	Monte Genzana – Alto Gizio Regional Nature Reserve	13,942786	41,94632	30/06/2021	Hair
RA3	Collarmele (AQ)	Sirente–Velino Regional Natural Park	13,622576	42,05434	07/03/2022	Muscle + Hair
RA4	Fucino Plain, Trasacco (AQ)	Bordering Sirente–Velino Regional Natural Park	13,580634	41,97543	22/07/2022	Hair
RA5	Pettorano sul Gizio (AQ)	Monte Genzana – Alto Gizio Regional Nature Reserve	13,942786	41,94632	07/10/2022	Hair
RA6*	Pettorano sul Gizio (AQ)	Monte Genzana – Alto Gizio Regional Nature Reserve	13,923531	41,92815	25/10/2020	Hair
RA7*	Pettorano sul Gizio (AQ)	Monte Genzana – Alto Gizio Regional Nature Reserve	13,942786	41,94632	31/05/2021	Hair

*Note:* ‘Sample name’ refers to the alphanumeric identification code assigned to each sample. ‘Municipality’ refers to the sampling location; ‘Protected areas and parks’ indicates the protected area within or near which the sampling coordinates (longitude and latitude) are located. ‘Longitude’ and ‘Latitude’ are reported in decimal degrees. ‘Date’ indicates the collection date, and ‘Sample type’ identifies the biological material collected. All listed samples yielded successful mtDNA‐ND5 sequencing, except those marked with an asterisk (*).

All tissue and hair samples were subsequently used for mtDNA analyses.

Detailed information on sample identity, municipality, protected area, geographic coordinates, sampling date, and sample type is provided in Table 2.

The main protected areas in which these new EWC occurrences were recorded are briefly described below to provide their geographical and ecological context:

- Abruzzo, Lazio, and Molise National Park (ALMP), established in 1922, which covers approximately 50,000 ha, with an additional 80,000 ha buffer zone. Forests account for about 60% of the park area, predominantly composed of beech. The park includes extensive mountain grasslands, limestone massifs, deep canyons, and some of the oldest beech forests in Europe, and functions as a major ecological corridor along the Apennine chain.
- Simbruini Mountains Regional Park (SMP), which covers 29,990 ha at elevations between 408 and 1075 m above sea level (a.s.l.) and hosts several Apennine species of high conservation value.
- Sirente–Velino Regional Natural Park, a mountain protected area characterised by extensive karst landscapes, high‐altitude grasslands, and well‐preserved beech forests. The park supports a high level of biodiversity and provides key habitats for several endemic and conservation‐priority species.

#### 2.2.2 Spatial Characterisation

To extract the 23 predicted occurrence probabilities from the Maxent model, a circular buffer was generated around each biological sample's location. The buffer radius approximated the mean home‐range size of EWCs in central Italy, as reported by Anile et al. (2017), and was calculated as the arithmetic mean of male (6.53 km^2^) and female (4.78 km^2^) home ranges. This corresponds to a radius of 1.34 km, rounded to 1.35 km (corresponding to a home range of 5.73 km^2^). Overlapping buffers were not merged. Following the genetic analyses of the biological samples (see below), differences in predicted occurrence probability among sample categories were assessed using pairwise Wilcoxon rank‐sum tests, with *p*‐values adjusted for multiple comparisons using the Benjamini–Hochberg procedure.

#### 2.2.3 Biological Samples

The sample set comprised 23 individual cats, represented by a total of 24 biological specimens (Table 2): seven hair samples and 17 tissue samples (muscle or skin). For one individual (RA3), both a hair and a muscle sample were available and analysed to maximise genotyping success. Thus, although both specimens from RA3 were sequenced, this individual was included only once in the subsequent analyses.

Hair samples were collected through non‐invasive monitoring. Following Anile et al. (2010), a 2.5 km grid was established to guide camera‐trap placement. Sixteen grid cells were selected, and hair traps were installed 1–3 m in front of the camera traps. Hair traps consisted of untreated wooden poles wrapped with Velcro and treated with *Valeriana officinalis* tincture. Hair samples were collected biweekly using sterilised tweezers and stored in paper envelopes until being sent to the lab for DNA extraction.

Tissue samples derived from carcasses (muscle or skin) of phenotypically EWC‐like individuals were collected by park rangers and classified according to Ragni and Possenti (1996). Tissues were preserved in 70% ethanol at −20°C and stored at the laboratories of the IZL dell'Abruzzo e del Molise (Teramo, Italy).

#### 2.2.4 Genetic Analyses

The DNA was extracted from all specimens using the Quick‐DNA Miniprep Plus Kit (Zymo Research). A fragment of the mitochondrial MT‐ND5 gene, widely used in felid phylogenetics due to its low homoplasy and absence of nuclear mitochondrial pseudogenes (numts) (Lopez et al. 1994; Driscoll et al. 2011), was amplified, enabling direct comparison with previous studies on Italian wildcats (Velli et al. 2023). The 835‐bp fragment corresponding to positions 13149–13983 of the domestic cat mitochondrial genome (*Felis_catus*_9.0; ENSFCAG00000032074.1) was amplified using primers F2B (5′‐TGCCGCCCTACAAGCAAT‐3′) and R3B (5′‐TAAGAGACGTTTAATGGAGTTGAT‐3′) (Velli et al. 2023). PCR products were sequenced by Sanger sequencing (Eurofins Genomics).

Sequence processing was performed in MEGA X (Kumar et al. 2018). Sequences were aligned, trimmed to positions 13243–13911 (final length 669 bp), and used to construct an mtDNA‐ND5 dataset. Haplotypes were identified using DNAsp 6.12.03 (Rozas et al. 2017) and classified following Velli et al. (2023). The taxonomic identity of sequences obtained from the hair samples was assessed by BLAST (https://blast.ncbi.nlm.nih.gov) and comparison with previously described *Felis silvestris* and *F. catus* haplotypes (Velli et al. 2023). Sanger chromatograms were also inspected for ambiguous or overlapping peaks at polymorphic sites as potential evidence of mixed mitochondrial templates.

Because mtDNA is maternally inherited and represents a single, non‐recombining locus, ND5 haplotypes were used to describe maternal lineage composition and were not considered sufficient to infer individual genomic admixture or distinguish historical introgression from contemporary hybridisation.

The final set of unique haplotypes was used for downstream analyses. Full protocols are provided in File S1.

All 21 occurrences with haplotype information, together with two occurrences lacking genetic data (RA6* and RA7*; Table 2), were mapped using the QGIS geographic information system (version 3.28.14).

## 3 Results

### 3.1 Model Outcomes

The species distribution model predicted several areas in Italy as highly suitable for the EWC. Comparison between the AUC value of the species distribution model (AUC_SDM_) and those of the null models (AUC_NM_) indicated that model performance was higher than expected under null models (AUC_SDM_ = 0.81, AUC_NM_ = 0.50, Figure S1).

**FIGURE 2:**
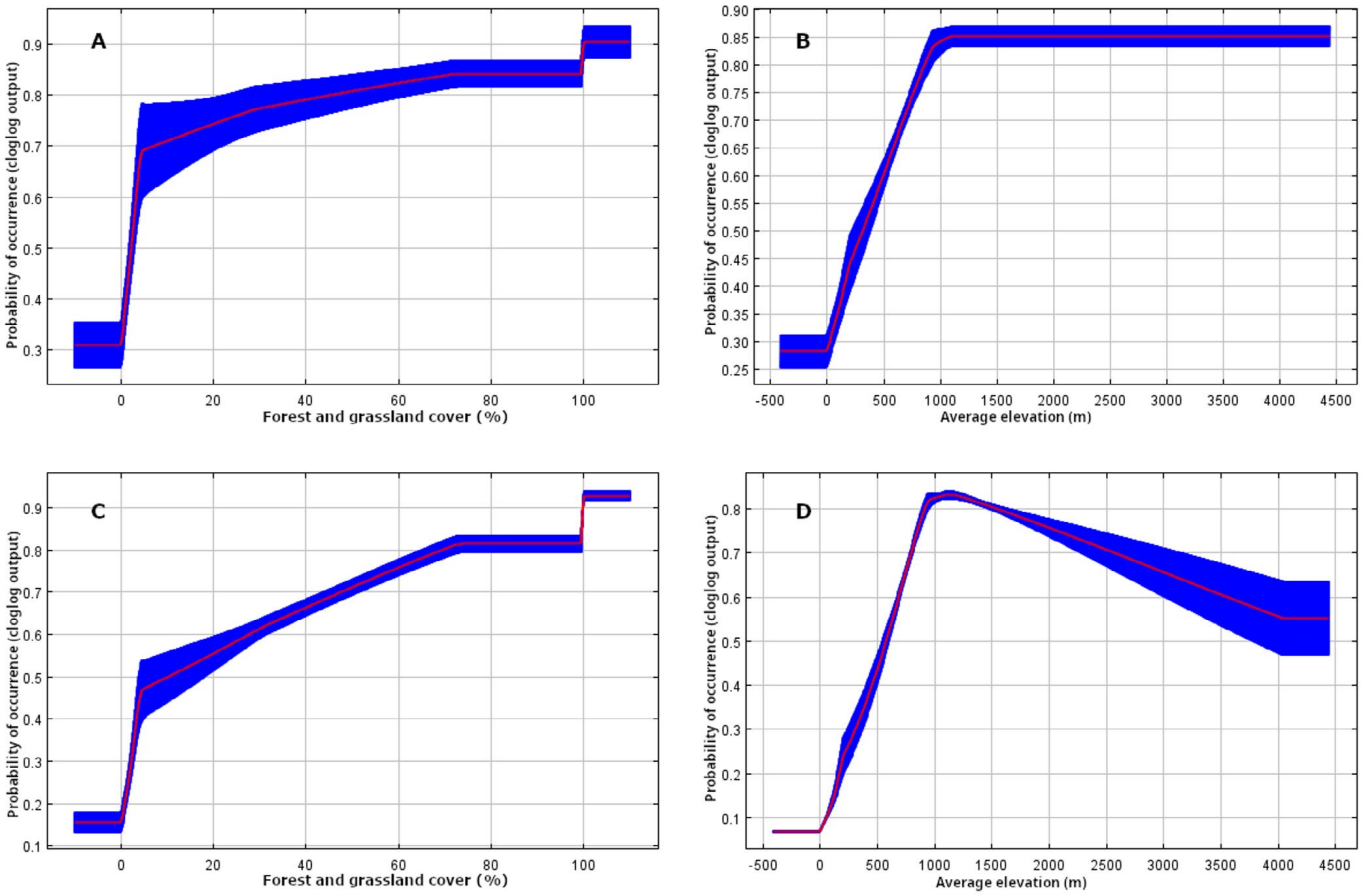
Response curves of predicted European wildcat occurrence probability (*P*_occ_) to forest and grassland cover and average elevation. (A) Marginal response to forest and grassland cover and (B) marginal response to average elevation, with all other environmental predictors held at their mean values. (C) Response to forest and grassland cover and (D) response to average elevation, when each predictor was used alone. Blue areas represent plus or minus one standard deviation (±1 SD), calculated from multiple replicate runs.

Forest and grassland extent and average elevation were the environmental variables contributing most to the Maxent model (Table 3). Marginal response curves showed that *P*_OCC_ increased sharply with increasing forest and grassland cover and then increased more gradually, tending to level off at higher cover values (Figure 2, panel A). Similarly, *P*_OCC_ increased with average elevation up to approximately 1000 m a.s.l., beyond which the response levelled off (Figure 2, panel B).

**TABLE 3:** Relative contribution of environmental variables to the Maxent model.

Variable	Percent contribution	Permutation importance
Forest and grassland (L6_F)	62.5	78.6
Average elevation (ELE)	36.1	20.9
Fruit trees and olive groves (L4_FTO)	0.8	0.4
Pastures and agriculture (L3_PAG)	0.6	0.1
Continuous and discontinuous urban fabric (L1_UF)	0	0
Agro‐forestry (L5_AGF)	0	0
Watercourses and water bodies (L8_WCB)	0	0
Road and rail networks (L2_RR)	0	0
Scrubland and sparsely vegetated land (L7_SSV)	0	0

When predictors were considered individually, *P*_OCC_ showed a similar positive relationship with forest and grassland cover, tending to level off at approximately 70%–75% cover (Figure 2, panel C). In contrast, the response to average elevation was unimodal, with *P*_OCC_ peaking at approximately 1000 m a.s.l. and subsequently declining towards higher elevations (Figure 2, panel D).

**FIGURE 3:**
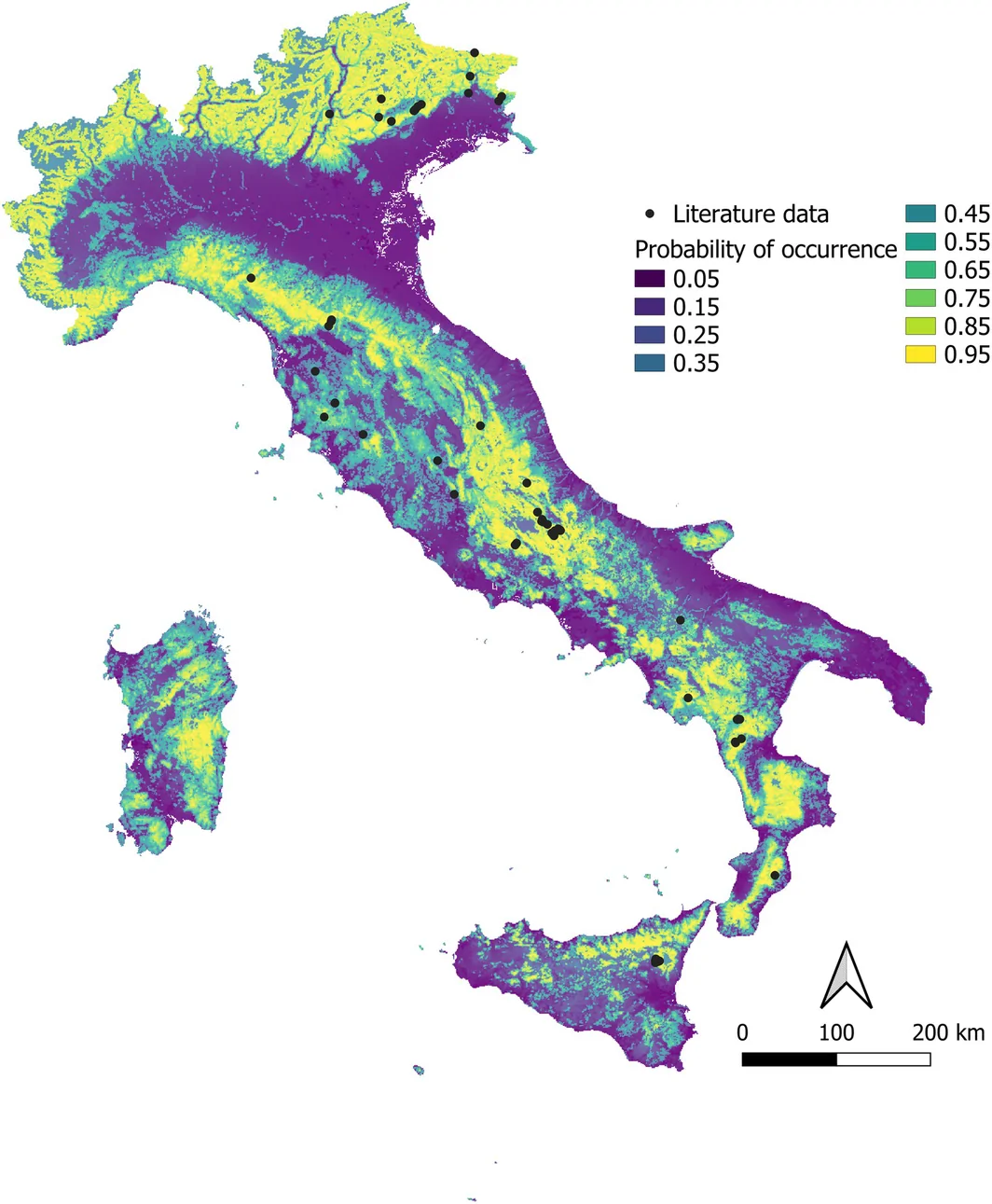
Predicted probability of European wildcat occurrence across the study area. The colour scale represents the predicted probability of occurrence using 10 classes at 0.10 intervals, ranging from 0.05 (dark purple) to 0.95 (yellow). Black points (literature data) show the 69 wildcat occurrence records used for model calibration, derived from three datasets: (i) camera‐trapping surveys and carcass recoveries from the ‘Progetto Gatto Selvatico’—https://www.museonaturalemaremma.it/gatto‐selvatico‐italia/; (ii) camera‐trapping records from the Abruzzo region collected by RA within the LIFE ESC360 project (LIFE17 ESC/IT/000001); and (iii) camera‐trapping records from Etna Regional Park, Sicily, reported by Anile et al. (2012).

The spatial distribution of *P*_OCC_ across Italy, together with wildcat occurrence records from the literature, is shown in Figure 3. Predicted probabilities of EWC occurrence, ranging from 0.05 (dark purple) to 0.95 (yellow), were higher along the Apennine chain and the Alpine region, which are characterised by forest areas and lower levels of human presence. Although lower, predicted suitability in lowland areas and coastal zones of the peninsula was not negligible.

### 3.2 Molecular Outcomes

The quality and quantity of DNA samples listed in Table 2 were assessed with spectrophotometric analysis (Tecan Infinite 200 PRO) prior to downstream analyses. Two hair samples yielded insufficient DNA and were therefore excluded from the genetic analyses (RA6* and RA7*). The remaining DNA samples were successfully amplified and sequenced. All sequences covered 835 bp of the MT‐ND5 gene and were then trimmed to 669 bp. The sequences obtained from hair and tissue samples belonging to the same carcass (RA3, Table 2) were identical and were therefore included only once in the analysis. All hair samples yielded interpretable mtDNA sequences, and no ambiguous or overlapping peaks indicative of mixed mitochondrial templates were detected in the Sanger chromatograms. The recovered haplotypes matched haplotypes previously described in European wildcats ( *Felis silvestris* ) or domestic cats ( *F. catus* ) (Velli et al. 2023). Alignment of the 669 bp sequences with the domestic cat reference sequence (NCBI: NC_001700; Table 4) revealed 11 single‐nucleotide variants (SNVs; 1.64%) and four haplotypes (d3, dw1, dw4, and w12), classified according to Velli et al. (2023). Under this classification scheme, the four haplotypes are grouped into three major haplogroups (d, dw, and w), previously reported as associated with domestic cats (d), shared among domestic cats, putative hybrids, and EWCs (dw), or reported in EWCs (w) (Velli et al. 2023). Among the 21 sequences analysed, haplotype frequencies were 71.4% for dw1, 14.3% for dw4 (both belonging to the dw haplogroup and accounting together for 85.7%), 9.5% for w12, and 4.8% for d3 (Table 4). It should be noted that the frequency of the dw1 haplotype may be overestimated, as three hair samples sharing this haplotype were collected at the same location within a short time frame (RA1, RA2, and RA5; Table 2). Although the camera trap images suggested the presence of different individuals, individual identity could not be genetically confirmed; therefore, the possibility that some of these samples originated from the same individual cannot be excluded.

**TABLE 4:** Single Nucleotide Variants (SNVs) identified in the 669 bp sequenced amplicons of the MT‐ND5 gene (mtDNA NADH dehydrogenase subunit 5) from 21 cats phenotypically identified as putative European wildcats collected in the Central‐Southern Apennines for this study (Table 2).

Position and variants on U20753.1 *Felis catus* mitochondrion complete genome (NCBI) ‐ MT‐ND5 (mtDNA NADH subunit 5)												
13248	13265	13285	13320	13326	13419	13534	13554	13611	13656	13679	13770	13899
A	T	T	A	G	G	G	A	**T**	**A**	G	A	C

Position on the 669 bp amplicon sequence													Haplotype ID	*N*.	%
6	23	43	78	84	177	292	312	369	414	437	528	657			
.	.	.	.	.	C	A	.	.	.	.	.	T	d3	1	4.8
.	.	.	.	.	C	A	.	.	.	.	.	.	dw1	15	71.4
G		C	G		C	A	.	.	.	.	.	.	dw4	3	14.3
.	C			A	C	A	C	C	G	A	G	.	w12	2	9.5
.	.	.	.										Total	21	

*Note:* Haplotype IDs follow Velli et al. (2023); *N*. indicates the number of individuals carrying each haplotype. Bold nucleotides indicate positions at which the reference sequence differs from all four haplotypes identified in this study. A dot (.) indicates that the nucleotide is identical to the corresponding nucleotide in the reference sequence.

### 3.3 Haplotype Distributions and Probability of Occurrences in the Central‐Southern Apennines

**FIGURE 4:**
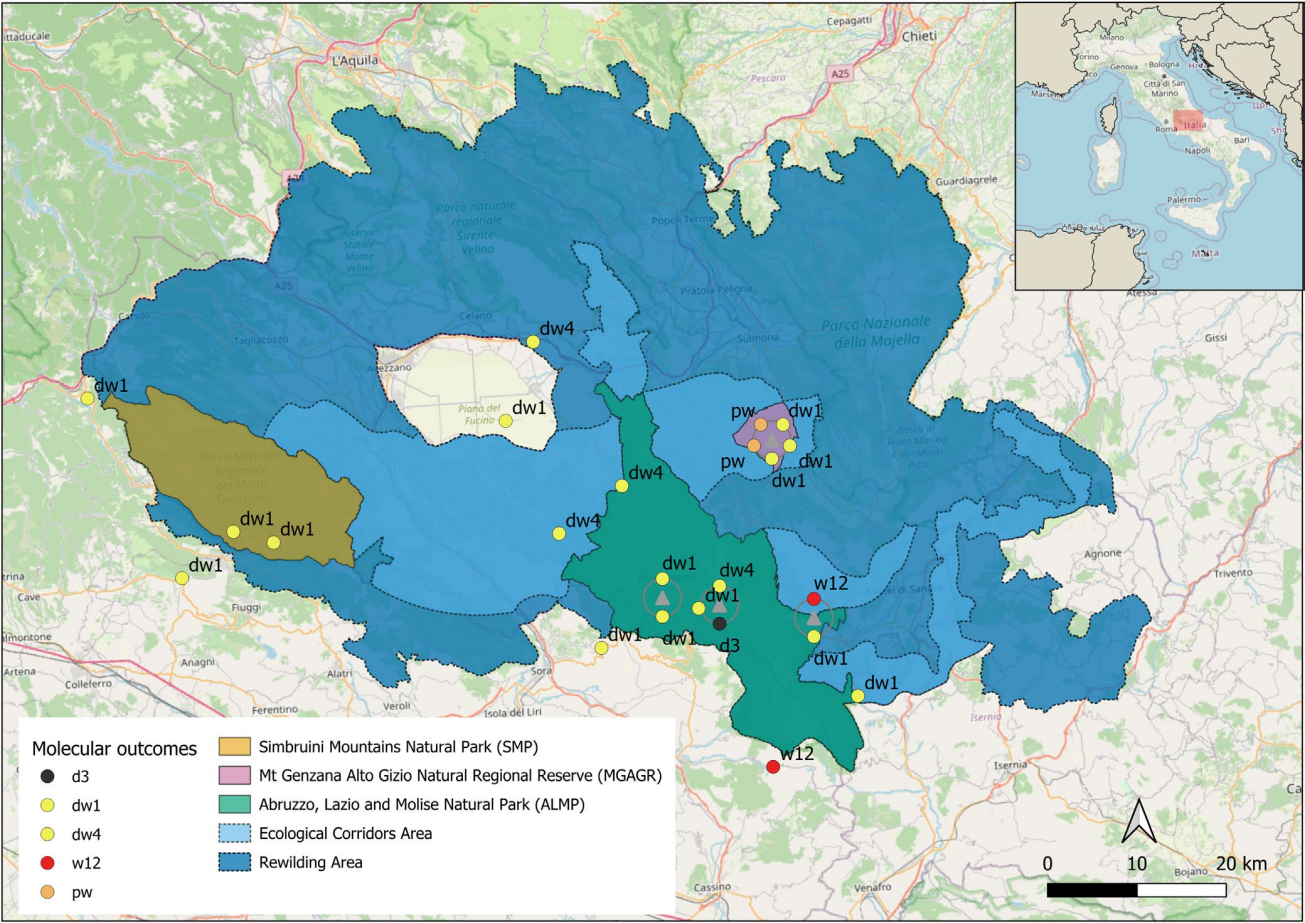
Geographical distribution of the *23 sampled individuals* in the Central‐Southern Apennines. *Twenty‐one individuals are shown according to their MT‐ND5* haplotypes (d3, dw1, dw4, and w12). The dw1 and dw4 haplotypes are both shown in yellow because they belong to the *dw* haplogroup, representing maternal lineages shared among European wildcats and domestic cats according to Velli et al. (2023). The two individuals for which no usable mtDNA sequence was obtained (RA6* and RA7*) are indicated as phenotypically wild (pw); this category therefore reflects phenotypic identification only and does not represent a molecular assignment. The boundaries of the protected areas, the Ecological Corridors Area, and the Rewilding Area are also shown. For clarity, labels associated with clustered sampling locations are displayed around their actual locations. The inset shows the location of the study area within Italy. Basemap: OpenStreetMap contributors, available under the Open Database Licence, obtained through the QuickMapServices QGIS plugin.

Ten of the 18 dw haplotypes were found primarily within the protected area boundaries (Regional Natural Park of the Simbruini Mountains, Abruzzo, Lazio and Molise National Park, Sirente–Velino Regional Natural Park, and Mount Genzana Alto Gizio Natural Regional Reserve—MGAGR), whereas the remaining eight were found in adjacent protected or ecological corridors. The two ‘w’ haplotypes were detected near the boundaries of ALMP (Figure 4).

**FIGURE 5:**
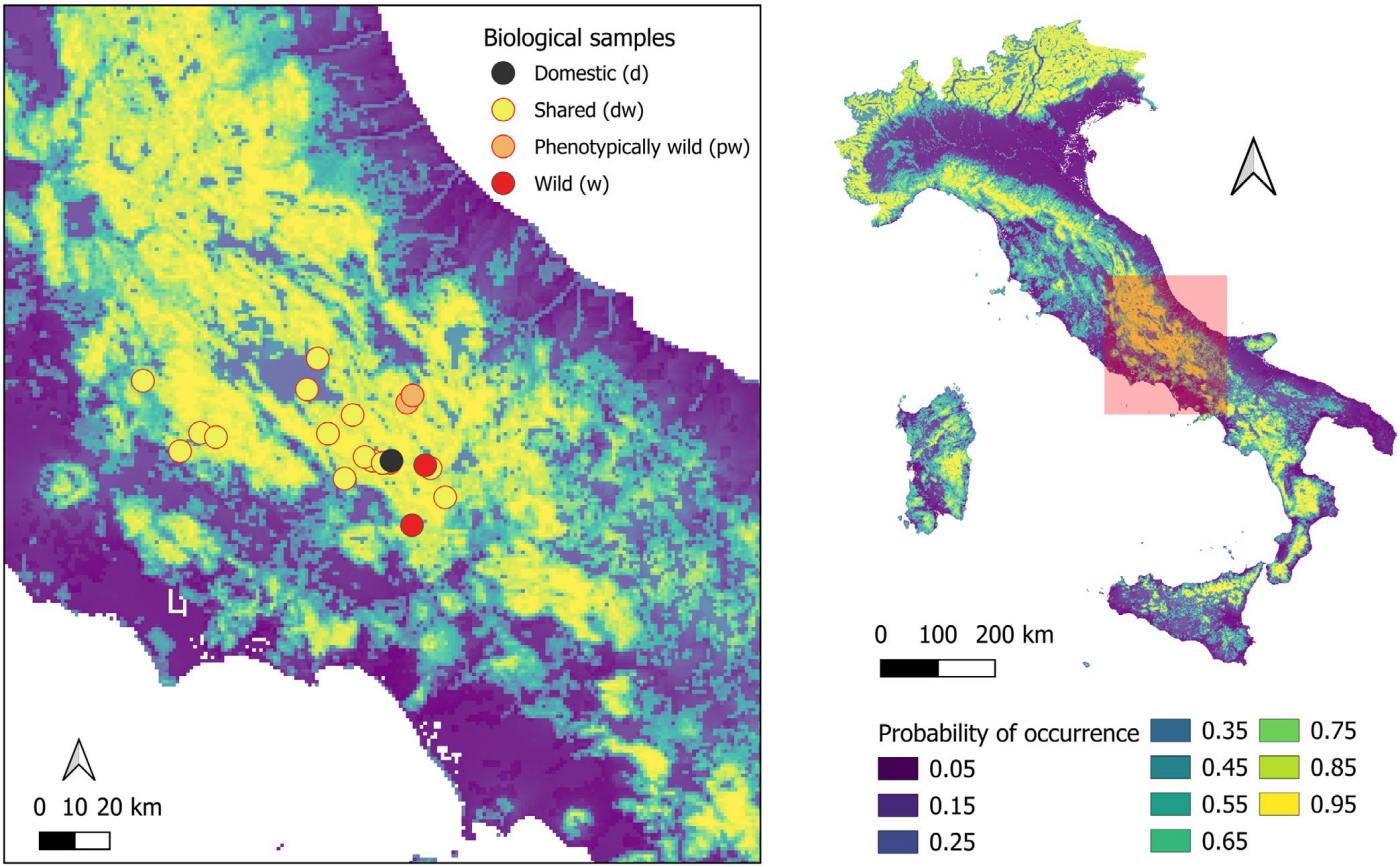
Predicted probability of European wildcat occurrence (*P*_OCC_) in relation to the locations of biological samples and their 1350 m buffers. The colour scale represents the predicted probability of occurrence using 10 classes at 0.10 intervals, ranging from 0.05 (dark purple) to 0.95 (yellow), as in Figure 3. Left: enlarged view of the focal area in the Central‐Southern Apennines. Dot colours represent the different biological sample categories: mitochondrial haplotype categories d, dw, and w, following Velli et al. (2023), as well as ‘Phenotypically wild’ (pw). Right: national prediction map showing the location of the focal area (red square).

When biological sample locations were overlaid on the prediction map, most samples occurred in areas with relatively high predicted probability of occurrence (Figure 5). This pattern is illustrated by samples RA6* and RA7*, which were collected within the boundaries of MGAGR, a relatively isolated area characterised by high predicted suitability for EWC. By contrast, only five samples (all carrying dw haplotypes) were located outside protected areas or ecological corridors and/or in areas characterised by high traffic intensity (samples SMP2, SMP3, RA3, RA4, ALMP8; Table 2).

**FIGURE 6:**
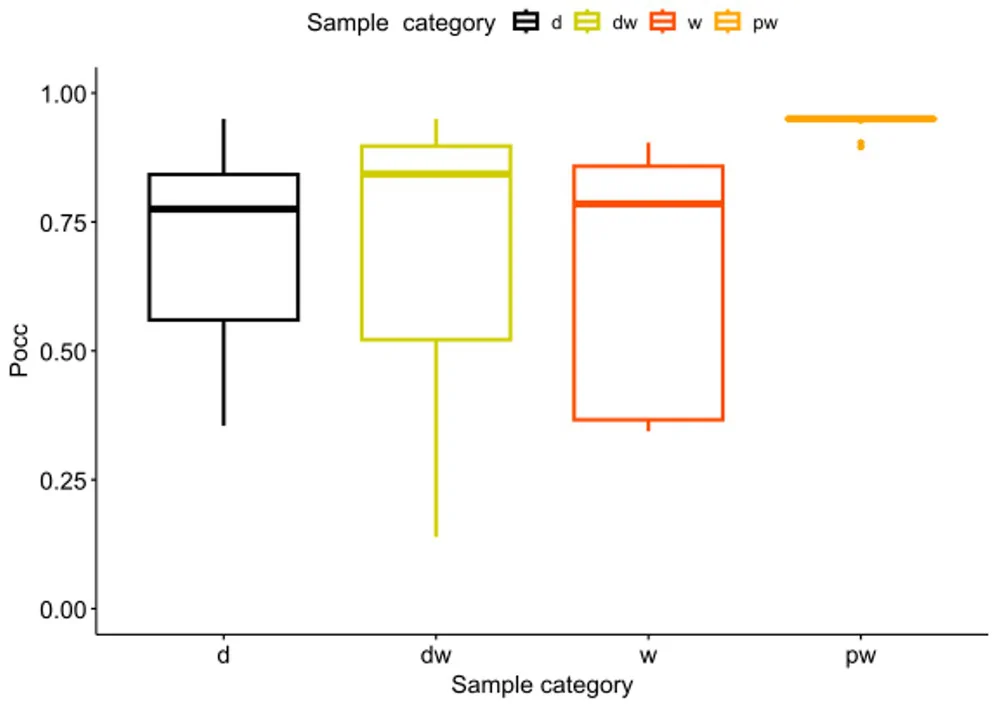
The predicted probability of occurrence (*P*_OCC_) within a 1350 m buffer around each biological sample location, grouped by haplotype categories and phenotypically wild (pw) type. On the horizontal axis (‘Sample category’), sample categories (see Table 4) are indicated as mitochondrial haplotype categories d, dw and w, following Velli et al. (2023), together with the additional category ‘phenotypically wild’ (pw). Boxes represent the interquartile range (IQR), encompassing the middle 50% of *P*_OCC_ values; the lower and upper bounds correspond to the first quartile (Q1) and third (Q3) quartile, respectively.

The distribution of predicted probabilities of occurrence (*P*_OCC_) within a 1350 m buffer around each biological sampling location, grouped by mitochondrial haplotype category (d, dw and w), and the phenotypically wild category (pw), is shown in Figure 6. Predicted occurrence probability differed among sample categories (Wilcoxon test with Benjamini‐Hochberg correction), with the pw category showing significantly higher values than d, dw, and w (all adjusted *p* < 0.001), and the dw category showing significantly higher values than w (adjusted *p* = 0.021). All other pairwise comparisons were non‐significant (adjusted *p* > 0.4).

## 4 Discussion and Conclusions

European wildcat populations exhibit locally expanding or declining trends driven by habitat suitability, prey availability, and human disturbance. Human activities, including urbanisation, intensive agriculture, and infrastructure development, fragment habitats into isolated patches, thereby reducing the species' area of occupancy and complicating detection and long‐term monitoring (Anile et al. 2019; Gil‐Sánchez et al. 2020; Vázquez García et al. 2024). Ecological niche modelling has contributed substantially to advancing our understanding of EWC distribution by generating spatially explicit predictions of habitat suitability under current environmental conditions, thus supporting conservation planning and management (Austin 2007; Phillips et al. 2009; Phillips et al. 2017). In this study, we developed an SDM based on phenotypic occurrence data to estimate, for the first time, the country‐wide likelihood of EWC occurrence in Italy. This model was designed to inform nationwide management by guiding monitoring efforts and prioritising targeted conservation actions.

Model performance, evaluated through AUC comparisons against null models, suggested good predictive performance (Raes and Ter Steege 2007; Bohl et al. 2019). Forest and grassland cover and elevation emerged as the dominant predictors of EWC occurrence, consistent with the ecological importance of structurally complex and relatively undisturbed habitats for the species (Anile et al. 2019; Ruiz‐Villar et al. 2023; Migli et al. 2025). Rather than reflecting an effect of elevation by itself, the association with higher average elevations may partly capture landscape conditions correlated with reduced human disturbance and more extensive natural habitats, as observed across the Apennine and Alpine regions. Conversely, lower suitability in intensively cultivated and densely populated areas, such as the Fucino Plain in the Central Apennines, is consistent with the sensitivity of wildcats to habitat alteration and fragmentation.

Within the Central Apennines, some of the highest predicted suitability values occurred within well‐preserved protected areas. This pattern is consistent with the main environmental relationships identified by the SDM, particularly the strong positive association with forest cover and, to a lesser extent, elevation. Protected areas in this region encompass extensive forested and mountainous habitats, which correspond closely to the environmental conditions associated with higher predicted suitability for the EWC. These areas may therefore play an important role in wildcat conservation by maintaining forest‐dominated habitats and limiting large‐scale land‐use transformation. However, because protected‐area status was not explicitly included as a predictor, this pattern should not be interpreted as evidence of a direct effect of protection by itself. More generally, the model did not explicitly incorporate quantitative anthropogenic pressure metrics such as habitat fragmentation or composite indices of human pressure. Although some anthropogenic land‐cover classes were represented within the LULC predictors, their effects may not fully capture the influence of human disturbance. This should be considered when interpreting habitat suitability patterns, and future models based on occurrence datasets should evaluate these additional predictors explicitly, provided that information is available.

The broad agreement between newly detected occurrences and predicted suitable areas provides additional support for the ecological consistency of the model, including the two phenotypically identified individuals excluded from genetic analyses (RA6 and RA7; Figure 5), whose locations were consistent with the habitat associations identified by the SDM.

At the local scale, integrating genetic sampling with complementary monitoring techniques enhances detection probability and provides insights into population structure and genetic diversity (Mattucci et al. 2013; Steyer et al. 2016; Viviani et al. 2024). While camera trapping proved effective, hair trapping using *Valeriana officinalis* tincture showed limited success. This result aligns with recent evidence indicating that alternative attractants, such as catnip, may be more effective for non‐invasive genetic sampling (Viviani et al. 2024).

Although all sampled individuals were phenotypically classified as wildcats, mitochondrial DNA analysis revealed a predominance of haplotypes shared with domestic cats: only 10% (ALMP9 and ALMP12) carried a haplotype (‘w’ type: w12) reported exclusively in wildcats (Driscoll et al. 2011; Velli et al. 2023). The w12 haplotype is particularly rare, as it was previously detected in only one individual among the broad European dataset analysed by Velli et al. (2023), a wildcat from Spain genetically assigned to *Felis silvestris* based on nuclear STR markers. In that study, w12 was identified as a private Iberian wildcat haplotype and was interpreted, together with other private haplotypes from the Iberian Peninsula, as potentially reflecting the historical isolation of wildcat populations south of the Pyrenees. Its detection in two individuals from the Central Apennines therefore extends the currently known geographic distribution of this rare maternal lineage beyond the Iberian Peninsula and adds to the mitochondrial diversity documented for Italian wildcats. However, given the maternal inheritance and single‐locus nature of mtDNA, the occurrence of w12 in Italy should not by itself be interpreted as evidence of a direct phylogeographic connection with Iberian populations.

The discrepancy between phenotype‐based identification and mitochondrial assignment also highlights the uncertainty inherent in morphological identification. Although diagnostic pelage traits provide a useful tool for distinguishing wildcats from domestic cats (Kitchener et al. 2005), phenotypic overlap with domestic cats and admixed individuals may result in occasional misclassification. Consequently, some uncertainty may affect the phenotype‐based occurrence records underlying the SDM and should be considered when interpreting habitat suitability patterns.

Recent genome‐wide evidence suggests that contemporary interbreeding between domestic cats and EWCs is limited despite long‐term sympatry across Europe (Jamieson et al. 2023). This broader genomic context is compatible with historical introgression contributing to the mitochondrial patterns observed here; nevertheless, our ND5 data alone cannot establish the timing or extent of admixture. Therefore, the observed haplotype composition should primarily be interpreted as evidence of shared maternal lineages rather than as a direct estimate of contemporary hybridisation. Nuclear or genome‐wide markers would be required to quantify admixture and distinguish recent hybridisation from historical introgression.

The genetic findings should also be interpreted in light of the relatively small sample size, as successful ND5 sequences were obtained from only 21 samples. Moreover, because individual identity could not be confirmed for all hair samples, potential non‐independence among samples may have biased haplotype frequency estimates, particularly dw1, and influenced their apparent spatial distribution. Therefore, the haplotype frequencies and spatial patterns reported here should be considered preliminary and interpreted cautiously.

At the national scale, the predicted distribution of suitable habitat showed a clear spatial pattern, with the highest suitability concentrated along the Apennine chain and the Alpine region, whereas lowland and coastal areas were generally less suitable. This pattern suggests that forested and grassland mountain systems represent the main areas of potential habitat for EWC in Italy, reflecting the combined influence of forest and grassland cover and the environmental conditions associated with elevation. The Apennine chain may therefore be particularly important for maintaining spatial continuity among suitable habitats across the peninsula, although functional connectivity was not explicitly assessed in our model. In the Central–Southern Apennines, the broad correspondence between newly detected occurrences and predicted suitable areas further supports the relevance of this region for EWC conservation and identifies protected and surrounding suitable habitats as priority areas for continued monitoring and habitat management.

Conservation strategies should therefore prioritise the preservation and restoration of forest and grassland across suitable elevational gradients, whereas maintaining or enhancing connectivity among suitable areas for wildcat populations. Although the extent and timing of wildcat–domestic cat admixture cannot be conclusively determined from mitochondrial data alone, the observed haplotype patterns provide relevant baseline information for genetic monitoring and highlight the value of complementing mtDNA with nuclear or genome‐wide markers in future studies.

Management strategies and awareness campaigns aimed at reducing potential wildcat–feral cat interactions, including responsible pet ownership and monitoring of feral cat populations, may therefore be considered within a precautionary conservation framework, particularly in areas of sympatry. Conservation planning could therefore consider buffer zones and ecological corridors around suitable and protected areas as potential measures to maintain landscape connectivity, particularly where habitat fragmentation may limit movement among suitable areas. In alignment with global biodiversity targets (e.g., protecting 30% of terrestrial and marine areas by 2030), integrative management approaches combining ecological modelling, genetic monitoring, and updated occurrence data through ongoing monitoring, supported by public engagement, provide a valuable framework for guiding conservation actions.

Overall, our findings highlight the value of integrating habitat‐suitability modelling, updated occurrence data, and genetic monitoring to improve knowledge of EWC distribution and maternal lineage composition in Italy. Continued monitoring, combined with broader genomic approaches, will be important for refining the spatial and genetic patterns identified here and providing a stronger evidence base for future conservation planning.

## Author Contributions

**Melissa Maini:** conceptualization (equal), data curation (equal), formal analysis (equal), investigation (equal), methodology (equal), resources (equal), visualization (equal), writing – original draft (equal), writing – review and editing (equal). **Chiara Polce:** conceptualization (equal), data curation (equal), formal analysis (equal), methodology (equal), software (equal), visualization (equal), writing – original draft (equal), writing – review and editing (equal). **Francesca Bernini:** data curation (equal), formal analysis (equal), methodology (equal), software (equal), visualization (equal), writing – original draft (equal), writing – review and editing (equal). **Maria Longeri:** conceptualization (equal), data curation (equal), funding acquisition (equal), investigation (equal), project administration (equal), supervision (equal), visualization (equal), writing – original draft (equal), writing – review and editing (equal). **Raffaella Milanesi:** data curation (equal), formal analysis (equal), methodology (equal), supervision (equal), writing – original draft (equal), writing – review and editing (equal). **Romolo Caniglia:** formal analysis (equal), methodology (equal), supervision (equal), writing – original draft (equal), writing – review and editing (equal). **Federica Mattucci:** data curation (equal), formal analysis (equal), visualization (equal), writing – original draft (equal), writing – review and editing (equal). **Edoardo Velli:** data curation (equal), formal analysis (equal), visualization (equal), writing – original draft (equal), writing – review and editing (equal). **Roberto Cazzolla Gatti:** conceptualization (equal), funding acquisition (equal), investigation (equal), resources (equal), supervision (equal), visualization (equal), writing – original draft (equal), writing – review and editing (equal).

## Funding

This research was supported by the University of Milan (Università degli Studi di Milano) through its departmental research funding programme, Line 2—Research Support Plan (Action A, 2023; grant number PSR2023_DIP_035).

## Conflicts of Interest

The authors declare no conflicts of interest.

## Supporting information


**Table S1:** Spatially explicit occurrence records of the European wildcat ( *Felis silvestris silvestris* ) used for calibration of the species distribution model (*n* = 69). The table reports sample ID, sampling location, geographic coordinates, phenotypic or genetic classification, data source, year and sampling methodology. MEP = Mount_Etna_Regional Park (CT); MGAGRNR = Monte_Genzana_Alto_Gizio_Regional_Nature_Reserve (AQ); ZLSNR = Zompo_Lo_Schioppo_Nature_Reserve (AQ), A_12 = Anile_et_al_2012; RA = Rewilding_Apennines; PGS = Progetto_Gatto Selvatico_Italy; CT = Camera_Trapping; OR = Opportunistic_Record.
**File S1:** PCR reactions were performed in a final volume of 10 μL containing 1 μL of genomic DNA (50 ng/μL), 1 μL of 10× PCR Gold Buffer (Applied Biosystems), 0.6 μL of MgCl₂, 0.8 μL of 0.2% bovine serum albumin (BSA), 0.09 μL of 10 mM dNTPs, 0.15 μL of each primer (10 μM), 0.2 μL of AmpliTaq Gold DNA Polymerase (5 U/μL), and molecular‐grade water to a final volume of 10 μL. PCR amplification consisted of an initial denaturation at 94°C for 15 min, followed by 50 cycles of denaturation at 94°C for 30 s, annealing at 55°C for 60 s, and extension at 72°C for 60 s, followed by a final extension at 72°C for 10 min.
**Figure S1:** Distribution of null‐model AUC values (AUC_NM_) obtained from 100 iterations (black distribution), shown together with the empirical AUC of the Maxent species distribution model (AUC_SDM_; red dot). Horizontal lines indicate the 0.01, 0.05, 0.50, 0.95, and 0.99 quantiles of the null‐model distribution. The empirical model AUC (AUC_SDM_ = 0.81) was higher than the median null‐model AUC (AUC_NM_ = 0.50).

## Data Availability

All data supporting the conclusions of this study are provided within the manuscript and its Supporting Information. Geographic coordinates and haplotype data are fully reported in the main text and tables to ensure transparency and reproducibility. The mtND5 sequences identified in this study were identical to sequences previously published in the sources cited in the manuscript. As no novel sequences were identified, no new sequence data have been deposited. The haplotype frequencies and distributions derived from these sequences are fully presented in the manuscript. All data required to evaluate the results are accessible to editors, reviewers, and readers.
