# Supplementary material for: Checking Wilderness Through Conservation Genetics: The Case of the European Wildcat in the Central Apennines, Italy
Source: Ecol Evol. 2026 Sep 24;16(9):e74418. doi: 10.1002/ece3.74418 (PMC13612743; doi:10.1002/ece3.74418)
Supplement: Supplementary file 1 [file ECE3-16-e74418-s001.zip › APPENDIX.docx]

**APPENDIX**

**Table_S1.** Spatially explicit occurrence records of the European wildcat (Felis silvestris silvestris) used for calibration of the species distribution model (n = 69). The table reports sample ID, sampling location, geographic coordinates, phenotypic or genetic classification, data source, year, and sampling methodology. MEP= Mount_Etna_Regional Park (CT); MGAGRNR= Monte_Genzana_Alto_Gizio_Regional_Nature_Reserve (AQ); ZLSNR= Zompo_Lo_Schioppo_Nature_Reserve (AQ), A_12= Anile_et_al_2012; RA= Rewilding_Apennines; PGS= Progetto_Gatto Selvatico_Italy; CT= Camera_Trapping; OR= Opportunistic_Record

| **Sample name** | **Protected areas and Parks/ Municipality** | **Longitude** | **Latitude** | **Sample category** | **Source** | **Temp. Coverage** | **Methodology** |
| --- | --- | --- | --- | --- | --- | --- | --- |
| **S1** | MEP | 14.921.997 | 37.735.034 | PW | A_12 | 2009 | CT |
| **S3** | MEP | 14.903.595 | 37.730.108 | PW | A_12 | 2009 | CT |
| **S5** | MEP | 14.893.534 | 37.734.597 | PW | A_12 | 2009 | CT |
| **S7** | MEP | 14.874.218 | 37.727.854 | PW | A_12 | 2009 | CT |
| **S8** | MEP | 14.887.123 | 37.741.630 | PW | A_12 | 2009 | CT |
| **S9** | MEP | 14.898.957 | 37.744.174 | PW | A_12 | 2009 | CT |
| **S10** | MEP | 14.886.225 | 37.727.136 | PW | A_12 | 2009 | CT |
| **S12** | MEP | 14.872.317 | 37.737.848 | PW | A_12 | 2009 | CT |
| **S13** | MEP | 14.878.425 | 37.757.413 | PW | A_12 | 2009 | CT |
| **S14** | MEP | 14.885.150 | 37.754.608 | PW | A_12 | 2009 | CT |
| **S17** | MEP | 14.904.222 | 37.737.031 | PW | A_12 | 2009 | CT |
| **S18** | MEP | 14.871.633 | 37.720.082 | PW | A_12 | 2009 | CT |
| **RA8** | MGAGRNR | 13.979.000 | 41.946.600 | PW | RA | 2020 | CT |
| **RA9** | MGAGRNR | 13.573.930 | 42.421.230 | PW | RA | 2020 | CT |
| **RA10** | MGAGRNR | 13.950.077 | 41.965.187 | PW | RA | 2020 | CT |
| **RA11** | ZLSNR | 13.389.651 | 41.828.396 | PW | RA | 2020 | CT |
| **RA12** | ZLSNR | 13.389.563 | 41.830.449 | PW | RA | 2020 | CT |
| **RA13** | ZLSNR | 13.408.796 | 41.845.965 | PW | RA | 2020 | CT |
| **RA14** | MGAGRNR | 13.752.851 | 42.049.909 | PW | RA | 2021 | CT |
| **RA15** | MGAGRNR | 13.974.787 | 41.952.756 | PW | RA | 2021 | CT |
| **RA16** | Secinaro (AQ), S.P. 11 “Sirentina” | 13.694.820 | 42.135.180 | PW | RA | 2021 | CT |
| **RA17** | Scanno_AQ | 13.892.733 | 41.900.224 | PW | RA | 2021 | CT |
| **RA18** | Olmo_di_Bobbi_Mountain_Pass_AQ | 13.741.387 | 42.046.046 | PW | RA | 2021 | CT |
| **RA19** | Scanno_AQ | 13.891.755 | 41.924.667 | PW | RA | 2021 | CT |
| **RA20** | MGAGRNR | 13.917.901 | 41.939.705 | PW | RA | 2021 | CT |
| **RA21** | Marsicana_Provincial_ Road_AQ | 13.753.731 | 42.051.261 | PW | RA | 2021 | CT |
| **RA22** | Marsicana_Provincial_ Road_AQ | 13.750.924 | 42.063.758 | PW | RA | 2021 | CT |
| **RA23** | MGAGRNR | 13.894.855 | 41.924.634 | PW | RA | 2021 | CT |
| **RA24** | MGAGRNR | 13.905.827 | 41.948.084 | PW | RA | 2021 | CT |
| **RA25** | Anversa_degli_Abruzzi_AQ | 13.814.977 | 42.012.723 | PW | RA | 2021 | CT |
| **RA26** | MGAGRNR | 13.894.855 | 41.924.634 | PW | RA | 2021 | CT |
| **RA27** | Marsicana_Provincial_ Road_AQ | 13.753.731 | 42.051.261 | PW | RA | 2021 | CT |
| **RA28** | Scanno_AQ | 13.865.977 | 41.927.685 | PW | RA | 2021 | CT |
| **RA29** | Goriano_Sicoli_AQ | 13.764.761 | 42.037.051 | PW | RA | 2022 | CT |
| **M1** | Radicondoli_SI | 11.103.193 | 43.248.381 | PW(C2) | PGS | 2009 | CT |
| **M2** | Radicondoli_SI | 11.103.323 | 43.251.103 | PW(C2) | PGS | 2010 | CT |
| **M3** | Cividale_del_Friuli_UD | 13.424.103 | 46.109.183 | PW(C2) | PGS | 2012 | CT |
| **M4** | Farra_d'Alpago_BL | 12.304.437 | 46.086.489 | PW(C2) | PGS | 2016 | CT |
| **M5** | Farra_d'Alpago_BL | 12.354.281 | 46.105.993 | W(C1) | PGS | 2017 | OR |
| **M6** | Cantagallo_PO | 11.068.955 | 44.038.749 | PW(C2) | PGS | 2017 | CT |
| **M7** | Cantagallo_PO | 11.073.860 | 44.052.012 | PW(C2) | PGS | 2017 | CT |
| **M8** | Trento_TN | 11.084.669 | 46.033.911 | PW(C2) | PGS | 2018 | CT |
| **M9** | Montale_PT | 11.034.202 | 43.992.947 | PW(C2) | PGS | 2018 | CT |
| **M10** | Caorera_BL | 11.934.518 | 45.951.807 | PW(C2) | PGS | 2019 | OR |
| **M11** | Cesclans_UD | 13.042.640 | 46.359.886 | PW(C2) | PGS | 2019 | CT |
| **M12** | Viggianello_PZ | 16.080.422 | 40.016.911 | W(C1) | PGS | 2020 | CT |
| **M13** | Orsomarso_CS | 16.020.366 | 39.796.322 | W(C1) | PGS | 2020 | CT |
| **M14** | Belluno_(BL) | 12.250.117 | 46.046.791 | PW(C2) | PGS | 2020 | CT |
| **M15** | Belluno_BL | 12.268.269 | 46.058.988 | PW(C2) | PGS | 2021 | CT |
| **M16** | Viggianello_PZ | 16.097.307 | 40.012.240 | W(C1) | PGS | 2021 | CT |
| **M17** | Sanza_SA | 15.471.524 | 40.256.608 | PW(C2) | PGS | 2021 | CT |
| **M18** | Paularo_UD | 13.117.851 | 46.583.542 | PW(C2) | PGS | 2021 | CT |
| **M19** | Stilo_RC | 16.385.972 | 38.479.761 | PW(C2) | PGS | 2021 | CT |
| **M20** | Mezzano_TN | 11.802.728 | 46.169.437 | PW(C2) | PGS | 2021 | CT |
| **M21** | Muris_UD | 13.011.110 | 46.197.771 | PW(C2) | PGS | 2022 | OR |
| **M22** | Sughera_FI | 10.848.599 | 43.558.347 | W(C1) | PGS | 2022 | OR |
| **M23** | Pulfero_UD | 13.467.982 | 46.151.420 | PW(C2) | PGS | 2022 | CT |
| **M24** | Viggianello_PZ | 16.062.985 | 40.011.911 | W(C1) | PGS | 2022 | Capture |
| **M25** | Torri_in_Sabina_RI | 12.629.380 | 42.340.469 | W(C1) | PGS | 2022 | OR |
| **M26** | Lacedonia_AV | 15.440.253 | 41.011.042 | PW(C2) | PGS | 2022 | CT |
| **M27** | Corniglio_PR | 10.001.807 | 44.460.318 | PW(C2) | PGS | 2022 | CT |
| **M28** | Monte_Cavallo_MC | 13.001.061 | 42.992.763 | PW(C2) | PGS | 2022 | OR |
| **M29** | Saracena_CS | 16.099.483 | 39.823.793 | W(C1) | PGS | 2022 | Capture |
| **M30** | Montenero_d'Orcia_GR | 11.465.809 | 42.942.413 | PW(C2) | PGS | 2022 | OR |
| **M31** | Saracena_CS | 16.023.246 | 39.788.491 | W(C1) | PGS | 2022 | Capture |
| **M32** | Orsomarso_CS | 16.020.167 | 39.798.986 | W(C1) | PGS | 2022 | Capture |
| **M33** | Massa_Marittima_GR | 10.960.164 | 43.116.640 | PW(C2) | PGS | 2023 | OR |
| **M34** | Rivai_BL | 11.761.723 | 45.994.565 | PW(C2) | PGS | 2023 | CT |
| **M35** | Avigliano_Umbro_TR | 12.427.131 | 42.670.854 | W(C1) | PGS | 2023 | OR |

**File_S1.** PCR reactions were performed in a final volume of 10 μL containing 1 μL of genomic DNA (50 ng/μL), 1 μL of 10× PCR Gold Buffer (Applied Biosystems), 0.6 μL of MgCl₂, 0.8 μL of 0.2% bovine serum albumin (BSA), 0.09 μL of 10 mM dNTPs, 0.15 μL of each primer (10 μM), 0.2 μL of AmpliTaq Gold DNA Polymerase (5 U/μL), and molecular-grade water to a final volume of 10 μL. PCR amplification consisted of an initial denaturation at 94°C for 15 min, followed by 50 cycles of denaturation at 94°C for 30 s, annealing at 55°C for 60 s, and extension at 72°C for 60 s, followed by a final extension at 72°C for 10 min.

**Figure_S1**. Distribution of null-model AUC values (AUC_NM_) obtained from 100 iterations (black distribution), shown together with the empirical AUC of the Maxent species distribution model (AUC_SDM_; red dot). Horizontal lines indicate the 0.01, 0.05, 0.50, 0.95, and 0.99 quantiles of the null-model distribution. The empirical model AUC (AUC_SDM_ = 0.81) was higher than the median null-model AUC (AUC_NM_ = 0.50).
