# Supplementary material for: Checking Wilderness Through Conservation Genetics: The Case of the European Wildcat in the Central Apennines, Italy
Source: Ecol Evol. 2026 Sep 24;16(9):e74418. doi: 10.1002/ece3.74418 (PMC13612743; doi:10.1002/ece3.74418)
Supplement: Supplementary file 1 [file ECE3-16-e74418-s001.zip › File_S1.docx]

**File_S1.** PCR reactions were performed in a final volume of 10 μL containing 1 μL of genomic DNA (50 ng/μL), 1 μL of 10× PCR Gold Buffer (Applied Biosystems), 0.6 μL of MgCl₂, 0.8 μL of 0.2% bovine serum albumin (BSA), 0.09 μL of 10 mM dNTPs, 0.15 μL of each primer (10 μM), 0.2 μL of AmpliTaq Gold DNA Polymerase (5 U/μL), and molecular-grade water to a final volume of 10 μL. PCR amplification consisted of an initial denaturation at 94°C for 15 min, followed by 50 cycles of denaturation at 94°C for 30 s, annealing at 55°C for 60 s, and extension at 72°C for 60 s, followed by a final extension at 72°C for 10 min.
